# Shared Decision Making and Choice for Bariatric Surgery

**DOI:** 10.3390/ijerph16244966

**Published:** 2019-12-06

**Authors:** Yi-Chih Lee, Wei-Li Wu

**Affiliations:** Department of International Business, Chien Hsin University of Science and Technology, Taoyuan 32097, Taiwan; wlwu@uch.edu.tw

**Keywords:** patient decision aids, shared decision making, bariatric surgery, patient preference, adult

## Abstract

The number of people undergoing bariatric surgery is increasing every year, and their expectations for surgery often differ greatly. The purpose of this study was to develop a patient-centered decision-making aid to help people define their weight loss goals and assist them in discussing their surgical treatment with surgeons. Before the operation, the patients were asked to read the shared decision-making text and conduct a self-assessment. After the operation, we evaluated the program using survey questionnaires. A total of 103 patients were formally included in this study. The results show that patients were very satisfied with the use of patient decision aids (PDAs), with a score of 4.3 points (±0.6), and the postoperative decision-making satisfaction was also very high, at 4.4 points (±0.5), while the postoperative regret score was low, at 1.6 points (±0.6). Their satisfaction with surgical decision making and decision regret were statistically significantly negatively correlated (*r* = −0.711, *p* < 0.001). The experience of using PDAs was statistically significantly negatively correlated with decision regret (*r* = −0.451, *p* < 0.001); the experience of PDA use was statistically positively correlated with decision satisfaction (*r* = 0.522, *p* < 0.001). Patient decision aids are a means of helping patients make informed choices before they seek to undergo bariatric surgery.

## 1. Introduction

The relationship between doctors and patients has always been a topic of concern in the medical domain, as poor communication between doctors and patients often impairs patient care, leads to important medical information being missed, delays treatment, hinders efficiency in diagnosis and treatment, and may lead to communication conflicts in serious cases [1]. Since medical behavior is a complex decision to be made by the public, more detailed information and considerations are needed [2]. In addition, as each person’s considerations are different, the benefits and disadvantages of treatment and medical uncertainty are also very different, according to personal feelings. Shared decision making by doctors and patients does not suggest, induce, or encourage the patient to choose, consent to, or follow a particular option, nor is it intended to replace medical staff’s instructions. Instead, it is intended to help patients make informed and value-based decisions together with the medical staff [3], and is a win–win decision-making approach for both doctors and patients.

In order to promote mutual respect and effective communication between doctors and patients, in 1997, Charles proposed the concept of shared decision making (SDM). In SDM, at least the doctor and the patient should participate together, where the doctor presents the empirical information of various dispositions, while the patient presents their individual preferences and values, and this information exchange and discussion will achieve the best possible treatment options [4]. In the process of medical communication, SDM is considered to be the best way to make decisions [5]. Patient decision aids (PDAs) can be in the form of leaflets, brochures, films, or web tools that provide a clear description of the various options, using comparisons to state possible benefits and harms and allowing patients to evaluate the impact of various good or bad conditions from their own perspectives, and then work with healthcare providers to make the most appropriate personal decision [6].

SDM is defined as a method in which clinicians and patients go through all stages of the decision-making process together and share protocols for treatment preferences and treatment options [7,8,9,10]. The form of SDM can be seen as a continuous band of two extremes—the “traditional medical model” and the “informed medical model”, as shown in Table 1 [7,11,12].

Patient-centered treatment [13] represents a new and important way to improve the quality of medical care. Patient autonomy is seen as a fundamental value [14], and the relationship between clinicians and patients has become a partnership [7]. In addition, in Europe, the World Health Organization emphasizes the need to involve patients in the development and legislation of medical care, and regulations to strengthen patient influence have been passed in many countries [15]. The King’s Fund “Making Shared Decision-Making a Reality” mentions that the applicable statements are that “there are multiple different options that will lead to different results”, “there is no clear right or wrong answer (decision)”, and “the correct decision must depend on the patient’s own specific needs and preset goals” [16]. Previous studies have sorted the appropriate and inappropriate situations for SDM, as shown in Table 2 [3].

O’Connor et al. mentioned that the effectiveness of decision aids in treatment or screening decisions includes improving patient health literacy, improving patient participation in medical decision making, increasing patient understanding of the advantages and disadvantages of different treatment options, promoting consensus between doctors and patients, increasing patient compliance with medical care, avoiding unnecessary surgery, avoiding the improper use of drugs, increasing patient satisfaction, improving the quality of medical care, and saving medical expenditures and expenses [17,18].

Therefore, SDM is a patient-centered clinical medical implementation process that combines the three elements of knowledge, communication, and respect. The goal is to enable medical staff and patients to share the existing empirical medical results before making medical decisions, provide all the options that patients can consider by combining the patient’s own preferences and values, reach a consensus on medical decision making, and support patients in making medical decisions that meet their preferences via the participation of both clinicians and patients in medical care [4].

The World Health Organization’s data on obesity guidelines show that the most important obesity-related diseases include diabetes, metabolic syndrome, gallbladder disease, dyslipidemia, dyspnea, and sleep apnea. The top 10 causes of death among the Taiwanese population are malignant tumors (e.g., colorectal cancer, breast cancer, endometrial cancer), heart disease, cerebrovascular disease, diabetes, chronic lower respiratory disease, chronic liver disease and cirrhosis, hypertensive disease, and chronic kidney disease, which are all related to obesity [19].

According to the definition of the World Health Organization (WHO) (2018), overweight and obesity are defined as abnormal or excessive fat accumulation that may impair health. For adults, the WHO defines that overweight is a BMI greater than or equal to 25; and obesity is a BMI greater than or equal to 30. For children aged between 5 and 19 years, overweight is a BMI-for-age greater than 1 standard deviation above the WHO Growth Reference median; and obesity is greater than 2 standard deviations above the WHO Growth Reference median. For children under 5 years of age, overweight is a weight-for-height greater than 2 standard deviations above the WHO Child Growth Standards median; and obesity is a weight-for-height greater than 3 standard deviations above the WHO Child Growth Standards median. In 2016, more than 1.9 billion (39%) adults aged 18 years and older were overweight (39% of men and 40% of women). Of these, over 650 million adults were obese. Overall, about 13% of the world’s adult population (11% of men and 15% of women) were obese in 2016. In 2016, an estimated 41 million children under the age of 5 years were overweight or obese. Over 340 million children and adolescents aged 5–19 were overweight or obese. The fundamental cause of obesity and overweight is that an increased intake of energy-dense foods that are high in fat; and an increase in physical inactivity due to the increasingly sedentary nature of many forms of work, changing modes of transportation, and increasing urbanization [20].

Weight loss methods include exercise, diet control, medication, and Chinese medicine treatment; however, for severely obese patients, such weight loss methods tend to have little effect in reducing weight. If the body Mass index (BMI) for obesity reaches 40 or more (so-called “morbid obesity”), the mortality rate is more than twice that of normal people; therefore, bariatric surgery is an effective treatment for morbid obesity [21]. According to the estimation of the American Society for Metabolic and Bariatric Surgery (ASMBS), in 2017, approximately 228,000 people worldwide accepted bariatric surgery [22]. According to the 2016 statistics of the Taiwan Society for Metabolic and Bariatric Surgery, about 2480 people sought obesity surgery in a year in Taiwan [23]. Bariatric surgery may have long-term psychological and social effects on patients, and while such surgery can improve the quality of life and appearance of patients [24], different weight loss surgeries are still accompanied by different sequelae [25].

For patients with morbid obesity, bariatric surgery can effectively reduce weight and maintain weight [26], and long-term tracking shows that it can effectively extend the life of the patient [27,28]. This invasive surgery can successfully reduce the weight of patients and improve their quality of life. It is attractive for patients. Early small-intestine bypass bariatric surgery is carried out to reduce nutrient absorption to achieve weight loss. In the 1980s, stomach partitioning surgery was invented in the United States, which separates the stomach into a large stomach and a small stomach, emphasizing the need to reduce the amount of food the patient needs to reach satiety in order to achieve weight loss. Bariatric surgery is a gastrointestinal surgery; thus, patients may have gastrointestinal tract discomfort after surgery, and there is a possibility of long-term undernutrition due to dietary bias. However, overall, the development of bariatric surgery has been an effective and long-lasting weight loss therapy [29]. Currently, mainstream bariatric surgery can be divided into the following three categories [29,30]:

1. BioEnterics Intragastric Balloon (BIB).

In this treatment, the surgeon puts a dry water balloon into the stomach of the patient through the gastroscope, and then injects an amount of physiological saline suitable for controlling the patient’s weight into the balloon. As the water balloon expands to create the feeling of existing food, the patient will feel their stomach as full and the body feels less hungry, which reduces food intake to achieve weight loss. BIB does not cause wounds on the patient’s body, and there is no need to use drugs; therefore, the patient can return home on the same day, which has little effect on the patient’s work. The longest time that the balloon can be placed in the stomach should not exceed six months in order to avoid the situation that the balloon is in place for too long and causes water leakage or intestinal obstruction. When a foreign body is initially placed into the patient’s stomach, a few patients may not be able to eat easily before the stomach becomes used to it; however, the main function of BIB is to change the eating habits of the patient, thereby forcibly guiding the patient to control the amount of food to achieve weight loss.

2. Restrictive procedures.

(1) Adjustable gastric banding surgery

Adjustable gastric banding surgery separates the stomach to create a small stomach of about 20 c.c. capacity above the stomach using an adjustable band and an adjustable outlet. The surgeon adjusts the width of the band with a syringe placed under the belly in order to achieve weight control by limiting the patient’s calorie intake. As the surgery does not change the configuration of the stomach, the risk is low. This kind of surgery requires high patient compatibility, long-term return visits, willingness to actively follow the doctor’s advice to reduce food intake, and appropriate exercise to achieve the maximum weight loss benefits of the surgery.

(2) Sleeve gastrectomy surgery

This surgical method cuts the stomach vertically from the greater curvature, and divides the stomach into a large stomach and a small stomach. The operation removes almost the entire bottom of the stomach. Thus, the secretion of ghrelin is greatly reduced, and the patient’s appetite is greatly lessened; therefore, the amount of food intake is also reduced to achieve the purpose of weight control. Sleeve gastrectomy surgery has become the new standard surgery for bariatric surgery in Asia. In addition to effective weight control, it has the effect of improving type 2 diabetes. Moreover, for the doctor, the operation is relatively simple as compared to other procedures, the learning curve is short, and patient safety is higher after surgery, thus, it is becoming the most frequently adopted bariatric surgery in Taiwan.

(3) Gastric plication

Also known as greater curvature plication, this operation sutures the intestine with a non-absorbable suture at the greater curvature. After the operation, the stomach becomes a narrow tubular stomach. As surgeons were not skilled in this procedure when this surgery was first performed, it caused patients to violently vomit after surgery; however, due to the cost of surgery, after the year 2000, Iranian surgeons improved this surgical procedure. Now, it is a kind of bariatric surgery that is usually combined with sleeve gastrectomy surgery or adjustable gastric banding surgery.

3. Mixed procedures

(1) Roux-en-Y gastric bypass surgery has been implemented worldwide for many years, is now the most widely performed weight-loss surgery, and is considered to be the gold standard. However, as it is extremely difficult to perform, the doctors must undergo a steep learning curve and medical teams must provide more experienced care. Hence, it is a potentially risky operation.

(2) Mini-gastric bypass surgery is also known as single anastomotic gastric bypass surgery. Taiwan performs the highest number of this surgery, and the effect is excellent. This operation keeps the gastrointestinal anastomosis away from the esophageal opening, thus avoiding bile reflux to the esophagus. This procedure is safer and simpler than Roux-en-Y gastric bypass surgery.

(3) Duodeno–jejunal bypass with sleeve gastrectomy (DJB + SG) is a duodenal transposition surgery that combines the advantages of both procedures for Asians and for those with lighter weight. This surgery has high complexity, difficulty, and risks, and has recently been developed to treat diabetes.

The public’s cognitive expectation of the “successful weight loss” of bariatric surgery is often unclear. The decision to choose which bariatric surgery is based on preference [31]; thus, the right choice depends on personal preference rather than general treatment principles. Treatment decisions made without first clarifying the preferences and values of each patient often lead to communication barriers between patients and medical professionals. Often, patients and medical professionals have different recognition of the success of weight loss and the weighting factors that regain health [32]. Although people can make surgical choices through surgical case comparisons, professional medical staff can use their clinical experience to provide patients with more important factors to consider after surgery, including appearance, impact on life, etc. PDAs can provide information on all possible treatment options and help patients clarify the importance of these treatments [33]. PDAs can improve patients’ knowledge regarding treatment options and risk perception, and the use of PDAs can cause patients to make decisions that are more in line with their expectations [34]. If people can have realistic expectations of the weight loss that bariatric surgery can achieve, then patient dissatisfaction with the medical outcome and the chance of a medical dispute will decrease. At present, reports on the use of PDAs for bariatric surgery are lacking and limited. Thus, in order to achieve the above objectives, this paper describes the development of Taiwanese PDAs for bariatric surgery, which includes patient satisfaction in the tool and their surgery choice after using PDAs.

## 2. Methods

### 2.1. Case Hospital Selection

To develop a PDA for bariatric surgery, the development process was designed according to NHS England, the Ministry of Health and Welfare in Taiwan, and the Summary Guide of Shared Decision Making, as recommended by the British Obesity and Metabolic Surgery Society (BOMSS) [35,36]. The process involved two weight loss and metabolic surgery clinicians, and three bariatric surgery case managers (including nurses and dietitians). The main considerations for selecting representative case hospitals for PDA development were: (1) The hospital must have a care team for weight loss and metabolic surgery. In addition to the surgeons, the team must include case managers, nutritionists, endocrinologists, and psychiatrists to jointly provide patients with various preoperative and postoperative care. (2) According to the American Society for Metabolic and Bariatric Surgery, the most common bariatric surgery procedures are gastric bypass, sleeve gastrectomy, adjustable gastric band, and biliopancreatic diversion with a duodenal switch [37]; thus, the surgeons of the selected case hospital must have at least the skills to perform these four bariatric surgeries. (3) The hospital must have many years of experience in bariatric surgery, in order that the medical team has long-term follow-up experience in tracking patients.

### 2.2. PDA Development and Design

**Figure 1:**
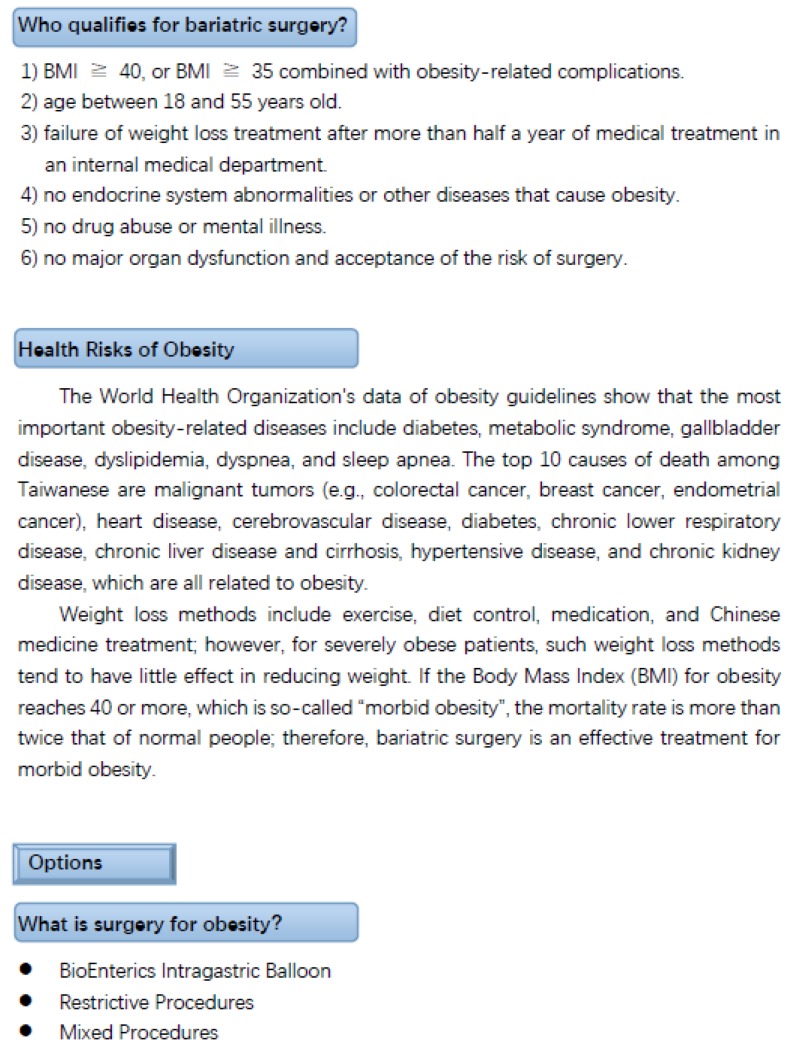
Patient decision aid.

In the first phase of this study, the content of the bariatric surgery decision aid was developed through in-depth interviews with a medical team. The PDA for weight loss surgery was designed in accordance with the outline recommended by the Ministry of Health and Welfare in Taiwan [36] and Healthwise Staff [38]. Therefore, medical staff followed this outline to discuss the PDA. The development of PDA tools for bariatric surgery included several steps [4]. Step 1: Explain the disease, treatment plan, and possible options to the patient, including an introduction of the disease and the harm caused to the body by being overweight, the weight loss program, the suitable target, and conditions of the operation (see Figure 1). Step 2: Provide comparative information of all treatment options for patient reference. Introduce the types of bariatric surgery currently approved in Taiwan, including restrictive and malabsorption surgery. Use pictures to show various surgical procedures, the risk of each operation, as well as the physical changes after surgery, adjustments to eating habits after surgery, etc. Step 3: Understand the patient’s preference for treatment options, understand what the patient cares about, the extent of care, and the individual’s treatment preferences. Step 4: Analyze the pros and cons of treatment options by comparing the advantages, disadvantages, risks, and side effects (complications) of each option, as well as the possible costs for the patient to consider. Step 5: Support patients in making medical decisions based on their values. Finally, the doctor, the nurse, and the patient again discuss the precautions for bariatric surgery, and decide on the treatment. The content of the PDA tool was revised and discussed several times by the team members, including the most common treatment options for bariatric surgery in the case hospital, and the tool text was presented in the language of the patient’s understanding. The finalized PDA was reviewed and approved by the Institutional Review Board of the case hospital (MSIRB: 2018019) for implementation.

### 2.3. Patients and Procedures

The patients came from a regional teaching hospital in Northern Taiwan. The bariatric surgeries performed by this hospital include BioEnterics Intragastric Balloon (BIB), adjustable gastric banding surgery, sleeve gastrectomy surgery, gastric plication surgery, mini-gastric bypass surgery, Roux-en-Y gastric bypass surgery, and duodeno–jejunal bypass with sleeve gastrectomy. Before the operation, the patient went to the bariatric and metabolic surgery center of the case hospital for pre-operative inquiry. First, the patient was required to read the PDA text content, and then the case manager discussed the tool content with the patient item by item, clarified the patient’s thoughts, and answered the individual case questions. Finally, the patient entered the outpatient area and communicated with the surgeon again regarding the bariatric surgery treatment options to determine the treatment options. The study time was from January 2019 to August 2019. After informed consent from the patients was obtained, a total of 103 effective bariatric surgery patients were collected.

### 2.4. Questionnaires

There are three questions in the PDA for cognitive measurement of bariatric surgery [39]. The questions are: “After weight-loss surgery, I will be able to eat normal amounts of food”, “Having weight-loss surgery can cause problems, but my being very overweight can also cause health problems”, “Surgery may be an option for me because my BMI is higher than 40”. There are three options of [True], [False], and [I’m not sure] in the answers.

After the use of PDA texts, the patients’ SDM experience was measured, and the items in the SDM Plan of the Joint Commission of Taiwan [40] were cited, with a total of 10 items. Patients were asked: “SDM can help me make the most suitable medical choices.” “SDM will help you better understand the advantages and disadvantages of various medical options currently faced”, “SDM helps you know more about what you are most concerned about when facing medical options”, etc. A Likert scale was adopted, with 5 points for strongly agree and 1 point for strongly disagree. The higher the score, the better the agreement with the use of the SDM tool (Cronbach’s α = 0.921). The design of 6 questions by Holmes-Rovner et al. (1996) [41] was used for the measurement of patients’ satisfaction with their bariatric surgery decisions, and a Likert scale was employed. The higher the score, the better the satisfaction with the decision quality (Cronbach’s α = 0.960). The scale designed by Brehaut et al. (2003) [42] with five questions was used as the decision-regret scale, where strongly disagree was indicated by 5 points and strongly agree was indicated by 1 point. There were also two reverse questions, where the higher the score, the greater the regret (Cronbach’s α = 0.867).

### 2.5. Data Analysis

Statistical analyses were performed using the Statistical Package for the Social Sciences (version 21.0 IBM SPSS Inc., Chicago, IL, USA) [43]. Descriptive statistics were analyzed by statistical methods such as mean and standard deviation. The Cronbach α coefficient of the scale was used to understand the internal consistency of the scale. Chi-square test, the Student’s *t*-test, ANOVA, correlation, and regression were conducted to examine the contribution and significance of the variables and dependent variables.

## 3. Results

### Patient Characteristics

Before the operation, 129 patients were asked to participate in the SDM plan. After removing one patient who did not receive bariatric surgery and 25 patients who failed to complete the follow-up questionnaire after surgery, 103 patients were formally included in the study. Among them, 64 were women (62.1%), 39 were men (37.9%), and the average age was 36.3 years (±10.8). There were 41 (39.8%) patients with sleeve gastrectomy surgery, 58 (56.3%) with mini-gastric bypass surgery, and 4 (3.9%) with Roux-en-Y gastric bypass surgery. The average BMI was 40.6 (±7.2). There were 10 (9.7%) diabetic patients. The average BMI for men was 42.8, which was higher than that of women at 39, and was statistically significant. In addition, 23.1% of the male patients had symptoms of diabetes, which was also much higher than the 1.6% of female patients, and with statistically significant differences. Table 3 shows the basic characteristics of the samples.

The reasons for choosing bariatric surgery were: the average score of [I have tried diet, exercise, and drugs, but none is effective] was 2.5 points; the average score of [My weight is bothering me very much, so I am willing to accept surgery, even if there is a risk] was 2.7 points; the average score of [I am confident that I can make a significant change in diet and exercise after surgery] was 2.5 points; the average score of [I am not worried about the cost of this surgery] was 2.4 points. Regarding the patients’ literacy of bariatric surgery, there were 72 people (69.9%), 43 women and 29 men, who gave correct answers to all questions, thus, the proportion of men with the correct answers was higher than that of women (74.4% vs. 67.2%, *p* = 0.511). The question with the highest wrong answer rate for patients was [After weight-loss surgery, I will be able to eat normal amounts of food]. The questions with high “I am not sure” ratios were regarding [postoperative eating habits] and [conditions for having bariatric surgery], and each was selected by four people. Before the discussion with the doctor, making decisions with confidence to implement bariatric surgery was about 4.5 points (sd = 0.9, range: 1–5).

Next, a questionnaire survey was conducted with the post-operation patients who performed the PDA, including their experience of using PDA, their satisfaction with the surgical decision making, and their decision regret. The results show that patients were very satisfied with the use of the PDA, with a score of 4.3 points (±0.6), and the postoperative decision-making satisfaction was also very high, at 4.4 points (±0.5), while the postoperative regret score was low, at 1.6 points (±0.6). Among them, their satisfaction with surgical decision making and decision regret were statistically significantly negatively correlated (r = −0.711, *p* < 0.001). The experience of using PDA was statistically significantly negatively correlated with decision regret (r = −0.451, *p* < 0.001); and the experience of PDA was statistically positively correlated with decision satisfaction (r = 0.522, *p* < 0.001) (Table 4). Women’s satisfaction with the experience of PDA use and surgical decision making were higher than men’s, and the score of decision regret was lower than that of men; however, there was no statistically significant difference. There was no statistically significant difference in the educational level of PDA experience, surgical decision satisfaction, or decision-regret scores.

Most of the decisions regarding bariatric surgery were made by patients accompanied by their parents, with 38 people (36.9%), including 21 women (55.3%) and 17 men (44.7%). There were 22 patients (21.4%) who made the decision themselves, including 10 males (45.5%) and 12 females (54.5%). Secondly, 18.4% of the patients (19 people) made the decision together with their spouse, including nine women (47.4%) and 10 men (52.6%). There were eight (7.8%) people whose decisions were made by the child or the spouse of the child. There were 16 (15.5%) people, including 14 women (87.5%) and two men (12.5%), whose decisions were made with another person. The higher the education level, the more decisions were made with the help of parents (45.1%, 23). The lower the education level, the more dependent they were on children or their spouses to make decisions (33.3%, 4). Thus, education levels and decision makers had a statistically significant relationship (*p* = 0.004). Table 5 shows the use of the patient decision aid. Table 6 shows the relationship between decision maker and education.

## 4. Discussion

Obesity affects patient behavior, quality of life, and productivity, and even shortens lifespan by about 5–20 years [44]. The World Health Organization states that “obesity is a chronic disease, and obesity rates are not improved” [20,45]. Today, bariatric surgery is considered an effective strategy to maintain the weight loss effect, significantly improve obesity-related comorbidities, reduce obesity mortality, and improve the quality of life of patients; moreover, bariatric surgery has also been proven to be cost-effective [46]. While it is easy to say that patients should be allowed to make decisions themselves, practical implementation of this is difficult [47].

A previous study reported that their PDA was feasible and acceptable for use in routine clinical weight management encounters, and the majority planned to use the PDA in the future [48]. Weinstein et al. (2014) pointed out that patients may benefit from shared decision making which integrates patient values and preferences with current medical evidence to assist in the complex bariatric surgery selection process [49]. Therefore, this study described in detail the development of a PDA for patients with obesity, which helps them to choose the bariatric surgery option that is best for them. The development of this PDA is based on the needs of patients and medical personnel, and describes the obesity disease and the application of bariatric surgery in words that patients can understand, identifies the patients suitable for bariatric surgery, analyzes and compares the advantages, disadvantages, and risks of different bariatric surgeries, supplements text with various surgical images to make it easier for patients to understand the implementation of the operation, and then describes the effects of changes on the body after surgery, including changes in dietary habits, exercise habits, and changes in the skin. In addition, the PDA guides patients to think about their concerns and clarify their preferences by comparing the impacts of bariatric surgery. It further analyzes the degree of help of weight loss treatments other than bariatric surgery to patients, and finally, patients and surgeons select the appropriate bariatric surgery in accordance with the patient’s values.

In the PDA test of patients’ perceptions of bariatric surgery in this study, 17.5% (18 cases) of patients mistakenly believed that after bariatric surgery, they would be able to eat a normal amount of food, while 3.9% (four cases) of patients gave the answer of “I’m not sure”. Among them, females and patients with a medium level of education had a higher rate of wrong answers. Even if the PDA text has a special description regarding postoperative eating habits (for example, patients need to [have more meals, each with a small amount of food, after surgery: you can only eat a few ounces of food at a time as your new stomach can only hold a small amount of food]), there were still patients who did not think that they would need to change their eating habits after surgery. Therefore, the case manager and the doctor could specifically target the patients who provided wrong answers before surgery, in order to enhance the healthcare explanation and reduce any inconsistent understanding of eating habits after surgery. In addition, as many as 95% thought that bariatric surgery may cause some problems; however, because being overweight can also cause health problems, they sought medical assistance. However, about 10% of the patients did not understand the conditions for weight-loss surgery. Patients’ answers in the PDA can provide an important reference for medical staff to communicate with them.

Furthermore, the reason that patients chose bariatric surgery was that their weight diminished their quality of life; therefore, even though the patients knew the risks of surgery, in order to improve their quality of life, they were willing to accept surgery. Among all the items, the patients attached the least importance to the cost of bariatric surgery. The possible reason is that Taiwan National Health Insurance specifies that if an obese patient meets the following conditions: (1) BMI ≥ 40, or BMI ≥ 35 combined with obesity-related complications; (2) age between 18 and 55 years old; (3) failure of weight loss treatment after more than half a year of medical treatment in an internal medical department; (4) no endocrine system abnormalities or other diseases that cause obesity; (5) no drug abuse or mental illness; (6) no major organ dysfunction and acceptance of the risk of surgery, the patient only has to pay for medical consumables, thus, the costs for the ward balance and the health insurance part are not high [50]. If the patient has commercial insurance benefits, the cost is even lower, thus, the cost of bariatric surgery was the aspect of the least concern for patients.

The PDA survey showed that up to 36.9% of patients were helped by their parents to select the treatment. The survey also showed that the proportion of patients making decisions on their own was only 21.4%, meaning about 80% of patients needed the support and assistance of family or others to make the decision. Scholars have pointed out that a characteristic of China’s medical decision-making model is that, no matter whether the patient has the ability to act, or whether the patient actually participates in the decision-making process, medical decision making is considered to be made by the whole family, and the patient is part of the family. Each family naturally appoints a family member as a family representative to play a coordinating role between the doctor and the patient, including talking, negotiating, and signing with the doctor on behalf of the family. This representative certainly cannot make his or her own claim, meaning a joint decision must be made with the whole family in most cases, including the patient himself or herself [51]. The results of this study prove that most patients indicated that their family members had assisted in the decision regarding bariatric surgery. Therefore, when PDA is used by medical staff, in addition to informing the patient, it may be necessary to simultaneously explain PDA to the patient’s family. This is in contrast to Western culture, which emphasizes respect for autonomy and highlights the individual’s rights [52].

This study conducted a questionnaire survey on patients with PDA after surgery, and the results show that patients with bariatric surgery thought that the implementation of the shared decision making made them feel less apprehensive about bariatric surgery. At the same time, through the PDA tool, patients could better understand the advantages and disadvantages of various weight loss methods, as well as the contents of the medical methods. The results of this study are similar to the findings of Nota et al. (2016) [33], meaning that patients were extremely satisfied with the use of the PDA. In addition, this study investigated patients’ satisfaction with the surgical decision, as well as their decision regret. Patient satisfaction with the decision-making regarding the bariatric surgery was extremely high, and decision regret was low. Regarding the use of the PDA, the more people agreed with the use of the PDA, the higher their satisfaction with the choice of bariatric surgery, and the lower their decision regret. Therefore, the use of the PDA allows patients to fully understand the treatment options of various bariatric surgeries, and confirms their own weight loss preferences before surgery, which results in patients being more confident in their own surgical decisions.

However, some problems are also reflected in the process of using the PDA. In the process of investigation, this study found that those who were older or had lower education levels lacked the patience to read the PDA content, or expressed a failure to understand the PDA content, and had to be assisted by medical personnel in completing the reading and understanding of PDA tools. In contrast, young patients suggested completing the PDA on the Internet, which would enable patients to consider their options with family members in the comfort of their own home. Therefore, a network PDA version could be a future direction for the weight loss center, and artificial intelligence could also be added to facilitate PDA decision making prior to the hospital visit.

## 5. Implications for Practice

Encouraging medical staff to use this PDA could help them to understand public knowledge regarding weight-loss surgery, which would strengthen the education program; moreover, it would allow medical staff to understand the patients’ preferences, as well as their real needs, allowing doctors and patients to communicate more smoothly, reduce the inconsistent cognition of the patients, reduce postoperative medical disputes, and improve patients’ medical safety.

## 6. Conclusions

Patient decision aids are a means of helping patients make informed choices before they seek to undergo bariatric surgery.

## Figures and Tables

**Table 1:** Shared decision making (SDM) model for treatment method.

Decision-Making Models	Traditional Medical Model	SDM	Informed Medical Model
Doctor’s role	Initiative: Only explain the selected information to the patient and choose the treatment they think is best.	Initiative: Explain all information and treatment possibilities to the patient. Can recommend an option. Work with the patient to determine the treatment method.	Passive: Explain all the information and treatment possibilities to the patient. Withhold advice. Do not make any decisions.
Patient’s role	Passive: Accept the advice of the clinician. It is necessary to cooperate with the doctor during patient recovery.	Initiative: Receive all information. Have their own judgments about the hazards and benefits of treatment options. Discuss their preferences with the clinician. Determine the treatment with the clinician.	Initiative: Receive all information. Form their own judgment. The patient is free to choose from the options provided by the clinician and can determine the treatment on their own.
Information	One way (largely) Doctor → Patient	Two way Patient ↔ Doctor	One way (largely) Doctor → Patient
Discussion	Doctor alone or with other doctors	Doctors and patients (plus potential others)	Patient (plus potential others)
Who makes a decision?	Doctor	Doctor and Patient	Patient

**Table 2:** Scenarios that are applicable and not applicable to SDM.

Situation		Description
Applicable situation	1. There is no clear empirical medical conclusion, or the timing of SDM is appropriate, as suggested by clinical diagnosis and treatment guidelines.	1. Existing evidence does not present strongly recommended options. 2. If the advantages and disadvantages of the options are close (benefit, risk, difficulty, or cost), then patient preference is an important determinant.
	2. Patient values and preferences vary greatly (different choices).	1. The option has an effect or side effect that the patient cares about, such as possible significant physical or mental function, image change, or pain.
	3. The balance of benefits and risks depends on the patient’s actions.	1. For example: patient medication, continuous monitoring, and dietary compliance.
	4. Serious illness.	1. For example: serious life-threatening diseases, advanced stages of major chronic diseases, multiple and debilitating chronic diseases.
Not an applicable situation	1. The quality and conclusion of the evidence can provide strong suggestions, the advantages outweigh the disadvantages, and the patient value and preference are high.	1. This topic is suitable for direct execution, without the need for SDM with the patient. 2. Unless the patient has other considerations, this option does not need to be included in the discussion.

**Table 3:** Patient-related characteristics (*n* = 103).

Variables	Categories	Female Group (*n* = 64)	Male Group (*n* = 39)	*p*-Value
Age, years		35.6 ± 11.4	37.3 ± 9.9	0.489
Education, *n* (%)	Junior high school (inclusive) or below	9 (14%)	3 (7.8%)	0.291
	High school or vocational high school	27 (42.2%)	13 (33.3%)	
	University (inclusive) or above	28 (43.8%)	23 (58.9%)	
Operation methods, *n* (%)	Mini-gastric bypass surgery	33 (51.6%)	25 (64.1%)	0.187
	Roux-en-Y gastric bypass surgery	4 (6.2%)	0 (%)	
	Sleeve gastrectomy surgery	27 (42.2%)	14 (35.9%)	
Body mass index (BMI)		39.0 ± 6.5	42.8 ± 7.7	0.015 *
Patients with diabetes mellitus, *n* (%)		1 (1.6%)	9 (23.1%)	<0.001 *

Note: case number (percentage); mean ± standard deviation; * *p* < 0.05.

**Table 4:** The correlation among patient decision aid (PDA) use experience, decision satisfaction, and decision regret.

Variables	Mean (Standard Deviation)	PDA Use Experience	Decision Satisfaction	Decision Regret
PDA use experience	4.3 (0.6)	1		
Decision satisfaction	4.4 (0.5)	0.522 **	1	
Decision regret	1.6 (0.6)	−0.451 **	−0.711 **	1

Note: ** *p* < 0.01.

**Table 5:** Use of the patient decision aid (*n* = 103).

Variables	Categories/Range	Female Group (*n* = 64)	Male Group (*n* = 39)	*p*-Value
Correct answer rate	I will be able to eat normal amounts of food.	76.6% (49)	82.1% (32)	0.623
	Having weight-loss surgery can cause problems, but my being very overweight can also cause health problems.	92.2% (59)	100% (39)	0.154
	Surgery may be an option for me because my BMI is higher than 40.	87.5% (56)	89.7% (35)	0.731
Making decisions with confidence		4.4 ± 0.9	4.7 ± 0.7	0.067
PDA use experience		4.3 ± 0.6	4.2 ± 0.6	0.696
Decision satisfaction		4.4 ± 0.5	4.3 ± 0.5	0.316
Decision regret		1.6 ± 0.5	1.7 ± 0.6	0.640
Main surgical decision maker	Patient	18.8% (12)	25.6% (10)	0.015 *
	Spouse	14.1% (9)	25.6% (10)	
	Children or children-in-law	12.4% (8)	0% (0)	
	Parents	32.8% (21)	43.6% (17)	
	Other	21.9% (14)	5.2% (2)	

Note: percentage (case number); mean ± standard deviation; * *p* < 0.05.

**Table 6:** The relationship between decision maker and education.

	Maker	Patient	Spouse	Children or Spouse	Parents	Other	*p*-Value
Education							
Junior high school (inclusive) or below		16.7% (2)	8.3% (1)	33.3% (4)	16.7% (2)	25% (3)	0.004 *
High school or vocational high school		20% (8)	15% (6)	7.5% (3)	32.5% (13)	25% (10)	
University (inclusive) or above		23.5% (12)	23.5% (12)	2% (1)	45.1% (23)	5.9% (3)	

Note: percentage (case number); * *p* < 0.05.
